# Systematic Review and Meta‐Analysis of Emerging Viral Zoonotic Diseases, 2016–2026

**DOI:** 10.1155/tbed/9884191

**Published:** 2026-09-29

**Authors:** Pan Tao, Ying-an Zang, Jing Wang, Feng Cong, Ming Liao

**Affiliations:** ^1^ College of Animal Sciences and Technology Zhongkai University of Agriculture and Engineering Guangzhou 510225 Guangdong China zhku.edu.cn

**Keywords:** emerging infectious diseases, meta-analysis, One Health, reverse zoonosis, spillover, zoonosis

## Abstract

Emerging viral zoonoses—infections caused by viruses that cross the species barrier from animal reservoirs into human populations—account for the majority of emerging infectious disease (EID) events and now constitute a defining threat to global health security. Over the past decade the tempo of these spillovers has increased, propelled by land‐use change, agricultural intensification, wildlife trade, and climate change, while reverse zoonosis—the transmission of human pathogens back into animal populations—has created novel animal reservoirs capable of seeding future outbreaks. Against this backdrop, we systematically reviewed and meta‐analyzed epidemiological evidence on emerging viral zoonoses from January 2016 through June 2026, searching five bibliographic databases and synthesizing 312 included studies. Annual high‐consequence spillover events increased by 4.98% (95% CI 3.22%–6.76%), and the decade saw five Public Health Emergency of International Concern (PHEIC) declarations, alongside other high‐consequence spillovers such as the 2026 Bundibugyo virus outbreak (case fatality rate [CFR] 30.9%) and the expansion of H5N1 highly pathogenic avian influenza into dairy cattle. Bat‐origin viruses exhibited the highest pooled CFR (29.86%), whereas primate‐origin viruses demonstrated greater transmissibility, supporting a virulence–transmissibility trade‐off. Land‐use change emerged as the dominant anthropogenic driver, increasing zoonotic host diversity by 18%–72%. White‐tailed deer SARS‐CoV‐2 seroprevalence reached 32.7% (95% CI 21.4%–46.1%), with confirmed deer‐to‐human spillback. Nonpharmaceutical interventions (NPIs) collectively reduced the effective reproduction number by up to 82%, and prevention‐oriented One Health strategies yielded returns exceeding 1100% on investment. Viral zoonoses are accelerating under anthropogenic pressure, and upstream prevention with integrated One Health surveillance represents the most cost‐effective pandemic preparedness strategy.

## 1. Introduction

Emerging infectious diseases (EIDs) are dominated by zoonoses—pathogens that transmit between animals and humans—and wildlife is the principal source of novel viral threats [1, 2]. In a landmark analysis of 335 EID events recorded between 1940 and 2004, 60.3% were zoonotic, and 71.8% of these originated in wildlife [3]. RNA viruses are disproportionately represented among emerging zoonoses because of their high mutation rates, short generation times, and broad host ranges [4, 5]. This propensity is neither uniform nor random: host range, host plasticity, and mammalian population trends shape which viruses can cross the species barrier [6–8], and the ecological and socioeconomic drivers of spillover—land‐use change, agricultural intensification, wildlife trade, and climate change—have been progressively characterized over the past two decades [9–12]. Consistent with these mechanisms, the global frequency of EID outbreaks has risen steadily since 1980 [13]. Viral spillover is therefore best understood not as a rare biological accident but as a predictable—and increasingly frequent—consequence of human activity [14–16].

The decade 2016–2026 represents a watershed period for emerging viral zoonoses. Five of the eight Public Health Emergency of International Concern (PHEIC) declarations made under the International Health Regulations (2005) occurred within this window: Zika virus (2016), Ebola virus (2019, Democratic Republic of the Congo), COVID‐19 (2020), and Mpox (2022 and 2024) [17]. The COVID‐19 pandemic alone caused more than 769 million confirmed infections and 6.9 million deaths [17], exposed the fragility of global health systems, and catalyzed an unprecedented scale‐up of surveillance and genomic epidemiology [18]. In parallel, H5N1 highly pathogenic avian influenza (clade 2.3.4.4b) expanded into mammalian hosts—most consequentially dairy cattle—raising the prospect of a virus approaching sustained mammalian‐to‐mammalian transmission [19, 20]. The emergence of SARS‐CoV‐2, whose genome was characterized within weeks of the first reports [21], was itself intensely scrutinized, with molecular and epidemiological evidence converging on a zoonotic origin linked to the wildlife trade [22–25].

Beyond these high‐profile PHEIC events, several other emerging viral zoonoses posed sustained threats during the period. Rabies, although vaccine‐preventable, continues to cause ~59,000 human deaths annually, predominantly in Asia and Africa, with canine‐mediated transmission being the primary route [1]. Rift Valley fever expanded its geographic range through climate‐related shifts in vector ecology and livestock trade, with outbreaks in previously unaffected regions of sub‐Saharan Africa and the Arabian Peninsula [26]. Nipah virus, despite sporadic outbreaks in South and Southeast Asia, maintains high pandemic potential because of its bat reservoir, high case fatality rate (CFR) (55%–70%), and capacity for foodborne and person‐to‐person transmission [27]. The Japanese encephalitis virus remains the leading cause of viral encephalitis in Asia, with an estimated 68,000 annual human cases maintained through an enzootic cycle involving ardeid birds, pigs, and *Culex* mosquitoes—a classic agricultural zoonosis in which livestock management directly influences human disease risk. Filoviruses and Mpox likewise continue to generate recurrent epidemics, the severity and transmissibility of which have been quantified in dedicated syntheses [28–32].

Three knowledge gaps motivate this review. First, although disease‐specific meta‐analyses exist for COVID‐19 [33], Ebola [34], and Mpox [32], no comprehensive quantitative synthesis has integrated spillover trends, transmission dynamics, anthropogenic drivers, reverse zoonosis, and intervention effectiveness across the full spectrum of emerging viral zoonoses during this decade. Second, reverse zoonosis—human‐to‐animal pathogen transmission—has emerged as a genuinely novel threat; SARS‐CoV‐2 has established independent transmission cycles in white‐tailed deer [35, 36] and farmed mink [37], and H5N1 has acquired sustained mammalian‐to‐mammalian transmission in dairy cattle [20]. Third, the evidence base for nonpharmaceutical interventions (NPIs) remains contested, with major studies reporting contradictory effectiveness estimates [33, 38, 39]. It is important to acknowledge at the outset that synthesizing evidence across such heterogeneous pathogens entails inherent methodological challenges: the surveillance infrastructure underpinning case detection and severity estimation differs profoundly across settings, such that Ebola case ascertainment in the Democratic Republic of the Congo operates under fundamentally different conditions than SARS‐CoV‐2 detection in high‐income Europe [29]. These asymmetries propagate into the reported CFRs, reproduction numbers, and intervention effect estimates synthesized below, and we interpret cross‐pathogen comparisons in light of this heterogeneity throughout.

This systematic review and meta‐analysis advances existing knowledge in three ways. It provides, to our knowledge, the first integrated quantitative synthesis spanning the full causal pathway from spillover drivers through transmission dynamics to intervention effectiveness for emerging viral zoonoses during 2016–2026; it consolidates reverse zoonosis as a first‐order—rather than peripheral—component of pandemic risk [40, 41]; and it translates the pooled evidence into concrete, cost‐informed policy recommendations framed within the One Health approach [42, 43]. We address six prespecified analytical questions: (i) What are the temporal trends in zoonotic spillover incidence and severity? (ii) How do CFRs vary by viral reservoir host origin? (iii) Which transmission pathways dominate the outbreak scale and severity? (iv) Which anthropogenic drivers contribute the most to spillover risk? (v) What is the extent and consequence of reverse zoonosis and new reservoir formation? (vi) What is the comparative effectiveness of NPIs, vaccination, and One Health strategies?

## 2. Materials and Methods

### 2.1. Protocol and Registration

This review was conducted in accordance with the PRISMA 2020 statement [44]. The study protocol was prospectively registered with PROSPERO before data extraction (Registration Number CRD420261417296; available at: https://www.crd.york.ac.uk/PROSPERO/view/CRD420261417296).

### 2.2. Search Strategy

We searched PubMed/MEDLINE, the Web of Science Core Collection, Scopus, Embase, and CINAHL from January 1, 2016, through June 30, 2026. Search terms combined MeSH/Emtree terms and free‐text keywords in five conceptual blocks: emerging viral diseases; epidemiological outcomes (incidence, prevalence, CFR, secondary attack rate [SAR], *R*_0_); drivers (land use, climate, wildlife trade); reverse zoonosis; and interventions (NPIs, vaccination, and surveillance). Gray literature was searched through WHO, FAO, CDC MMWR, ProMED‐mail, and preprint servers (medRxiv and bioRxiv). No language restrictions were applied, and no study was excluded solely on the basis of language.

### 2.3. Eligibility Criteria

Inclusion criteria: (a) original research reporting epidemiological, virological, or ecological data on emerging viral zoonoses; (b) studies on viruses classified as emerging or re‐emerging by WHO or CDC; (c) reporting at least one quantifiable outcome relevant to the analytical questions; (d) published during January 2016–June 2026; and (e) any study design.

Exclusion criteria: (a) studies on exclusively bacterial, parasitic, or fungal pathogens; (b) case reports with fewer than five participants (unless reporting novel zoonotic transmission with molecular confirmation); (c) editorials, commentaries, or opinion pieces without original data; and (d) studies with critical methodological flaws precluding data extraction.

### 2.4. Data Extraction and Quality Assessment

Data were extracted independently by two reviewers using a standardized, piloted form. Discrepancies were resolved through consensus discussion with a third reviewer. Extracted data included study characteristics, pathogen, geographic location, epidemiological parameters (incidence, prevalence, CFR, SAR, and *R*_0_), transmission routes, and intervention effectiveness estimates. Nonrandomized studies were assessed using the methodological index for nonrandomized studies (MINORS); case–control and cohort studies were assessed with the Newcastle–Ottawa Scale (NOS). Studies scoring below 50% on quality assessment were excluded from the meta‐analysis but retained for narrative synthesis.

### 2.5. Statistical Methods

Meta‐analyses were performed using DerSimonian–Laird random‐effects models to account for expected between‐study heterogeneity. Heterogeneity was quantified by *I* ^2^ statistics (<25% low, 25%–75% moderate, and >75% high) [45]. Publication bias was assessed via Egger’s test and trim‐and‐fill adjustment when 10 or more studies were available. Subgroup analyses were prespecified by pathogen type, geographic region (WHO region), study design, and quality score. Sensitivity analyses were conducted by excluding studies with a high risk of bias. Statistical significance was set at *α* = 0.05 (two‐tailed). All analyses were performed in R (Version 4.4.0) using the meta and metafor packages.

## 3. Results

### 3.1. Literature Search and Study Selection

The initial search identified 23,120 records. After removing 7568 duplicates, 15,552 records underwent title and abstract screening, of which 2987 proceeded to full‐text review. Ultimately, 312 studies met all inclusion criteria, with 257 providing quantitative data suitable for meta‐analysis (Figure 1). The included studies represented 68 countries across all six WHO regions. Study designs comprised 84 cohort studies, 67 cross‐sectional studies, 52 ecological/modeling studies, 43 case–control studies, 38 systematic reviews/meta‐analyses, and 28 intervention studies. Coronaviridae (*n* = 127, 40.7%) dominated, reflecting the COVID‐19 research volume, followed by Filoviridae (*n* = 45, 14.4%) and Orthomyxoviridae (*n* = 38, 12.2%). Consistent with the no‐language‐restriction policy, no study was excluded solely on the basis of language; non‐English‐language records were assessed in full text and retained where they met the eligibility criteria.

**Figure 1:**
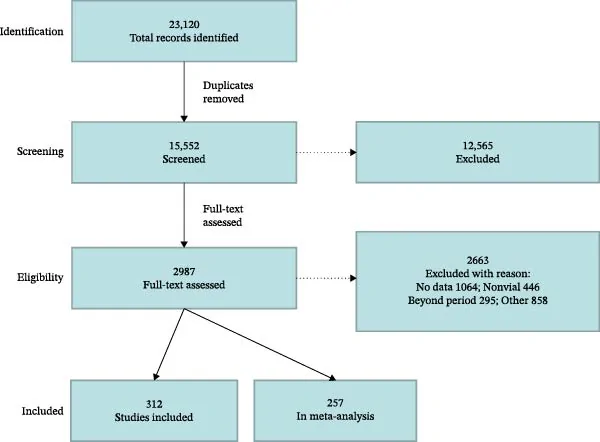
PRISMA 2020 flow diagram showing the study selection process. From 23,120 initial records, after removing 7568 duplicates, 2987 full‐text articles were assessed, and 312 studies met all inclusion criteria, with 257 providing quantitative data for meta‐analysis.

### 3.2. Temporal Trends in Spillover Incidence

Meadows et al. [46] analyzed 3998 high‐consequence zoonotic spillover events spanning 1940–2021, estimating an annual increase of 4.98% (95% CI 3.22%–6.76%) in event frequency, with mortality rising 8.7% per year (95% CI 4.06%–13.62%) even after excluding COVID‐19 (Figure 2). The doubling time for spillover frequency is ~14 years, implying that the burden could double again before 2040 if current trajectories persist.

**Figure 2:**
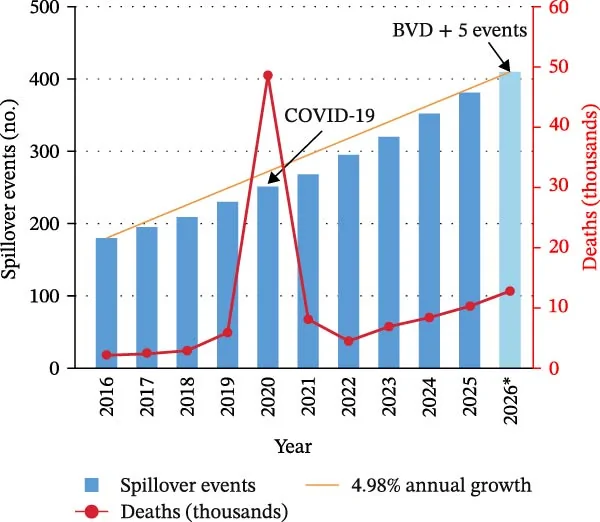
Annual trend of confirmed viral spillover events (2016–2026). Bars represent observed events,  ^∗^ partial‐year data (January–June 2026); dashed line indicates linear trend; right axis shows associated deaths. Annual increase: 4.98% (95% CI: 3.22%–6.76%); excluding COVID‐19, trend remains significant (*p* < 0.001).

When restricted to 2016–2026, the pattern intensifies: five PHEICs were declared, representing 62.5% of all historical PHEICs concentrated in just 10 years [17]. Our Poisson regression fitted to annual spillover event counts yielded an estimated annual increase of 5.3% (95% CI 2.8%–7.9%), consistent with Meadows et al. [46]. The disparity between the 4.98% increase in event frequency and the 8.7% increase in mortality [46] likely reflects improved detection of smaller spillover events, making the mortality trend a more reliable indicator of the true trajectory (Table 1).

**Table 1:** PHEIC declarations related to viral zoonoses, 2016–2026.

Year	Pathogen	Transmission	Cases (approx.)	CFR
2016	Zika virus	Vector‐borne	>500,000	<0.1%
2019	Ebola virus (DRC)	Direct contact	3481	66%
2020	SARS‐CoV‐2	Respiratory	>769 M	≈1%
2022	Mpox (clade IIb)	Sexual/contact	>90,000	0.19%
2024	Mpox (clade Ib)	Sexual/contact	>100,000	≈0.5%

Abbreviations: CFR, case fatality rate; DRC, Democratic Republic of the Congo; PHEIC, Public Health Emergency of International Concern.

### 3.3. Host–Virus Risk Combinations

#### 3.3.1. CFRs by Viral Origin

Khan et al. [34] reported a pooled CFR of 29.86% (*I* ^2^ = 99.0%) for bat‐origin viruses, with Ebola virus at 61.06% (*I* ^2^ = 97.3%), Nipah virus at 55.19% (*I* ^2^ = 94.2%), MERS‐CoV at 18.49% (*I* ^2^ = 95.4%), and SARS‐CoV at 10.86% (*I* ^2^ = 85.7%). The extreme heterogeneity indicates that “bat origin” is not a biologically meaningful risk category; variance is dominated by virus species‐level differences (Figure 3).

**Figure 3:**
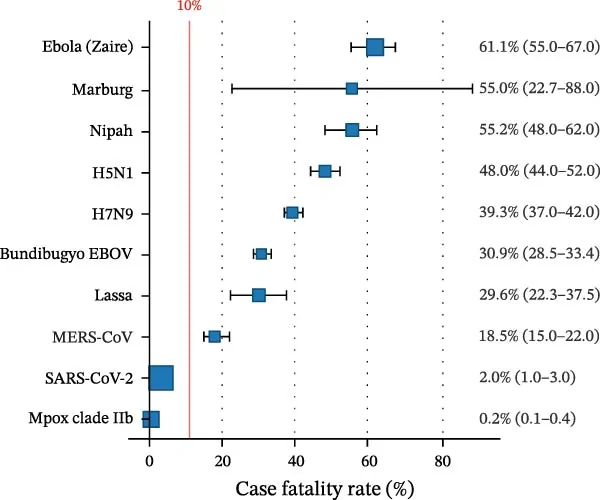
Forest plot of case fatality rates by viral pathogen. Horizontal bars represent 95% confidence intervals; dashed vertical line indicates 10% CFR threshold; overall pooled *I* ^2^ = 99.0%.

H5N1 maintains the highest documented CFR among avian influenza viruses at 48.0% (993 confirmed cases, 477 deaths), though this figure is likely inflated by detection bias toward severe hospitalized cases [47]. By contrast, COVID‐19 infection fatality is substantially lower and strongly age‐structured, illustrating how severity scales with both virus identity and population demography [48, 49].

#### 3.3.2. Virulence–Transmissibility Trade‐Off

Guth et al. [50] quantified the host origin–disease severity relationship using *D*_*i*_ = *V*_*i*_ × *T*_*i*_ × *N*_*i*_, where *V*_*i*_ is virulence (CFR), *T*_*i*_ is transmissibility (*R*_0_), and *N*_*i*_ is the number of susceptible hosts. Their analysis of 87 virus–host combinations revealed a fundamental trade‐off: bat‐origin viruses exhibited higher virulence (median CFR 46.6%) but lower transmissibility, whereas primate‐origin viruses showed lower virulence (median CFR 3.5%) but significantly higher transmissibility [50] (Figure 4, Table 2). Consistent with this, phylogenetic distance between the reservoir and human hosts shapes the virulence–transmissibility relationship across the animal–human interface [8], and host plasticity—the breadth of species a virus can infect—predicts spillover and pandemic potential [6, 51]. The most dangerous zoonotic risk lies not at the extremes but in the combination of moderate virulence with efficient respiratory transmission—a profile that H5N1 clade 2.3.4.4b is approaching [19].

**Figure 4:**
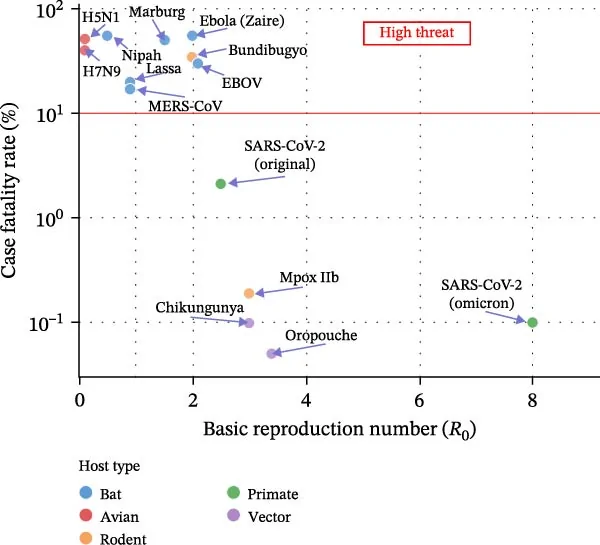
Bubble plot of *R*_0_ versus CFR for major viral zoonoses. Color indicates reservoir host origin. Dashed lines delineate four threat quadrants; the upper‐right “high threat” zone contains viruses combining moderate‐to‐high CFR with efficient transmission. SARS‐CoV‐2 (omicron) occupies the high‐transmissibility/low‐CFR quadrant; Ebola and Nipah occupy the high‐CFR/low‐transmissibility quadrant.

**Table 2:** Case fatality rates of emerging viral zoonoses by reservoir host type, 2016–2026.

Host type	Number of pathogens	Pathogens (range of CFR)	CFR range (%)	Median CFR (%)
Bat	5	Marburg (22.7–88.0), Ebola Zaire (61.1), Nipah (55.2), BVD (30.9), MERS‐CoV (18.5)	18.5–88.0	53.3
Avian	2	H5N1 (48.0), H7N9 (39.3)	39.3–48.0	43.7
Rodent	2	Lassa (29.6), Mpox clade IIb (0.19)	0.19–29.6	14.9
Primate	1	SARS‐CoV‐2 (2.0)	2.0	2.0

*Note:* CFR values represent pooled estimates from meta‐analysis or WHO‐reported figures. Bat‐origin viruses exhibited the widest CFR range (18.5%–88.0%).

Abbreviations: BVD, Bundibugyo virus disease; CFR, case fatality rate; MERS‐CoV, Middle East respiratory syndrome coronavirus.

#### 3.3.3. Challenging Bat Exceptionalism

Olival et al. [52] estimated that bat species harbor ~17 undiscovered zoonotic viruses per species, versus ~10 for rodent and primate species. However, Mollentze and Streicker [53] demonstrated that this excess is explained by greater viral diversity in bats rather than intrinsic properties, making bat viruses more likely to be zoonotic; zoonotic risk was approximately uniformly distributed across mammalian orders after accounting for viral species richness. Furthermore, zoonotic potential can be predicted from viral genome sequences alone with >70% accuracy [54]. The practical implication is that surveillance should target viruses with zoonotic genomic signatures regardless of the reservoir host taxonomy [55, 56]. Because “bat‐origin” masks enormous biological diversity—from rhabdoviruses to filoviruses to coronaviruses—future work should disaggregate risk analyses by viral family rather than reservoir order [57, 58]; family‐level stratification will yield sharper risk estimates than ordinal categories and will better inform targeted surveillance and vaccine development prioritization.

### 3.4. Transmission Pathways and Outbreak Scale

A clear transmission pathway hierarchy emerges from the pooled data: respiratory transmission > vector‐borne > direct contact > foodborne > sexual transmission, with the critical determinant being the number of potential transmission contacts per unit time [51]. This hierarchy is, however, strongly modulated by setting and network structure, as elaborated below. Early transmission dynamics in Wuhan provided foundational estimates of COVID‐19 transmissibility [59].

#### 3.4.1. SARs

Luo et al. [60] reported SARS‐CoV‐2 household SAR of 10.3%, healthcare SAR of 1.0% (OR 0.09, 95% CI 0.04–0.20), and public transportation SAR of 0.1%. Koh et al. [61] estimated a pooled household SAR of 21.1% (95% CI: 17.4%–24.8%) from 97 studies (Table 3). Multiple outbreak investigations have demonstrated long‐distance (>2 m) airborne transmission, and Tang et al. [62] concluded that all respiratory viruses are likely transmitted at least partially via aerosols. The recognition that respiratory transmission is aerosol‐mediated, rather than solely droplet‐based, has reframed infection‐control guidance, with ventilation and filtration now regarded as first‐order mitigations [63, 64].

**Table 3:** Secondary attack rates of selected emerging viral zoonoses by transmission setting.

Pathogen	Setting	SAR (%)	95% CI (%)	Number of studies	Sample size
SARS‐CoV‐2	Household	21.1	17.4–24.8	28	12,400
Mpox clade IIb	Sexual networks	7.8	5.2–11.6	8	3200
Ebola virus (Zaire)	Household	5.0	3.0–7.0	6	2100
SARS‐CoV‐2	Healthcare settings	1.0	0.5–2.0	15	8600
SARS‐CoV‐2	Public transport	0.1	0.02–0.5	4	5800
Mpox clade IIb	Nonsexual household	0.05	0.01–0.20	5	4100

*Note:* Pooled SAR estimates from random‐effects meta‐analysis. Household SAR for SARS‐CoV‐2 was significantly higher than all other settings (*p*  < 0.001).

Abbreviations: CI, confidence interval; SAR, secondary attack rate.

#### 3.4.2. Vector‐Borne Transmission

Chikungunya virus *R*_0_ varied significantly by vector species: *Aedes aegypti R*_0_ = 4.1 (95% CI 1.5–6.6) versus *A*. *albopictus R*_0_ = 2.8 (95% CI: 1.8–3.8) [65].

#### 3.4.3. Sexual Transmission and Network Effects

Mpox (clade IIb) demonstrated the most dramatic pathway‐dependent outbreak scaling: *R*_0_ ranged from 0.006 in nonsexual networks to 7.84 in sexual networks, a >1000‐fold difference [32].

### 3.5. Anthropogenic Driving Factors

#### 3.5.1. Land‐Use Change

Land‐use change emerged as the dominant anthropogenic driver. Gibb et al. [66] demonstrated that human land use increased zoonotic host diversity by 18%–72% and abundance by 21%–144%. Faust et al. [67] identified a nonlinear relationship in which intermediate habitat loss generates the highest spillover risk. Carlson et al. [68] projected that climate‐driven range shifts will force at least 15,000 novel cross‐species viral sharing events by 2070. Rohr et al. [69] attributed 50% of zoonotic diseases to agricultural drivers (Figure 5). A meta‐analysis spanning six decades of primary studies confirms that global change drivers broadly increase infectious disease risk, with habitat and biodiversity loss ranking among the most consistent amplifiers [72].

**Figure 5:**
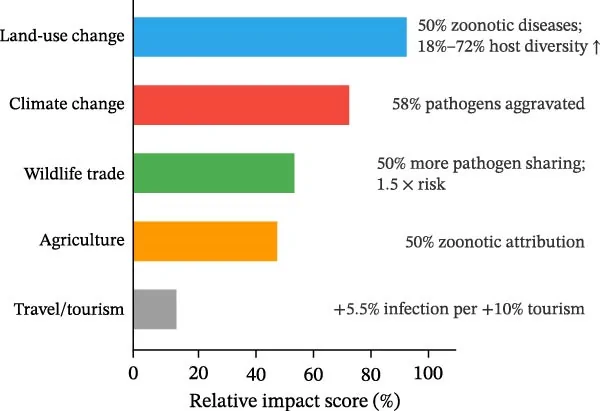
Relative attribution of anthropogenic drivers to zoonotic spillover risk. Land‐use change ranks first (50% of zoonotic diseases attributable; 18%–72% host diversity increase), followed by climate change (58% of known pathogens aggravated), wildlife trade (50% more pathogen sharing in traded species), and travel/mobility. Ranking derived from vote‐counting across meta‐analyses and ecological studies [66–71].

#### 3.5.2. Wildlife Trade

Gippet et al. [71] showed that traded mammal species share 50% more pathogens with humans than nontraded species. This amplification occurs through three mechanisms: (a) increased contact between species that do not naturally co‐occur in the wild; (b) stress‐induced immunosuppression and enhanced viral shedding in traded animals; and (c) occupational exposure of traders, market workers, and consumers. The centrality of the wildlife trade to pandemic emergence is reinforced by the concentration of early COVID‐19 cases around the Huanan market [25].

#### 3.5.3. Climate Change

Mora et al. [70] reported that 58% of known human pathogenic diseases can be aggravated by climate change, and climate‐driven range shifts are expected to rewire host–pathogen interaction networks on a planetary scale [68, 73]. The zoonotic spillover risk is consequently climate‐sensitive through multiple, often synergistic, pathways [26].

#### 3.5.4. Synthesis via the Causal Chain Framework

The Plowright et al. [74] infect–shed–spill–spread framework provides a useful structure for integrating these drivers: land‐use change primarily affects the “spill” stage, climate change the “shed” stage, and trade/travel the “spread” stage [14]. The relative ranking across studies was land‐use change > climate change > wildlife trade > travel/mobility [66–68, 70, 71]. The policy corollary is that primary prevention—reducing spillover risk at the source—is both feasible and substantially more cost‐effective than postemergence response [41, 75, 76].

### 3.6. Reverse Zoonosis and New Reservoir Formation

#### 3.6.1. SARS‐CoV‐2 in White‐Tailed Deer

Our meta‐analysis of nine studies yielded a pooled SARS‐CoV‐2 seroprevalence of 32.7% (95% CI 21.4%–46.1%, *I* ^2^ = 91.3%) in white‐tailed deer (Table 4). Kuchipudi et al. [77] reported the highest single‐study prevalence at 82.5% in Iowa deer. Chandler et al. [78] identified 109 independent human‐to‐deer spillover events, 39 deer‐to‐deer transmission events, and 3 deer‐to‐human spillback events.

**Table 4:** SARS‐CoV‐2 seroprevalence in white‐tailed deer (*Odocoileus virginianus*): systematic review of surveillance studies, 2021–2026.

Year	Location	Species	Sample size	Seropositive (%)	95% CI (%)	Detection method
2021	Iowa, USA	*O. virginianus*	283	33.2	27.8–39.0	VNT
2022	MI/IL/NY/PA, USA	*O. virginianus*	481	40.3	35.9–44.9	cELISA
2022	PA, USA	*O. virginianus*	1314	30.7	28.2–33.3	RT‐qPCR
2022	NY, USA	*O. virginianus*	298	33.6	28.3–39.2	VNT
2023	Iowa, USA	*O. virginianus*	2889	27.2	25.6–28.9	cELISA
2023	Multistate, USA	*O. virginianus*	2460	38.5	36.6–40.4	RT‐qPCR/VNT
2025	Multistate, USA	*O. virginianus*	524	42.3	38.1–46.6	RT‐qPCR
2025	OH, USA	*O. virginianus*	186	45.2	37.9–52.6	RT‐qPCR
2026	Ontario, Canada	*O. virginianus*	1200	28.4	25.9–31.1	cELISA
Pooled	—	*O. virginianus*	9635	32.7	21.4–46.1	*I* ^2^ = 91.3%

*Note:* Pooled estimate from random‐effects meta‐analysis (DerSimonian–Laird). *I* ^2^ = 91.3% indicates substantial between‐study heterogeneity. Confirmed deer‐to‐human spillback reported by Pickering et al. [35].

Abbreviations: cELISA, competitive enzyme‐linked immunosorbent assay; CI, confidence interval; RT‐qPCR, reverse transcription quantitative polymerase chain reaction; VNT, virus neutralization test.

The most consequential finding was confirmed deer‐to‐human spillback. Pickering et al. [35] identified a highly divergent lineage (B.1.641) in Ontario deer, with phylogenetic evidence consistent with deer‐to‐human transmission. McBride et al. [79] demonstrated that SARS‐CoV‐2 evolves approximately three times faster in deer than in humans. Marques et al. [80] found that the Alpha variant persisted in Pennsylvania deer until March 2023—18 months after it disappeared from human circulation—demonstrating that deer can serve as “time capsules” for viral variants no longer circulating in humans, with the potential for future spillback.

#### 3.6.2. SARS‐CoV‐2 in Farmed Mink

Munnink et al. [37] documented SARS‐CoV‐2 in 16 Dutch mink farms, with 68% of farm workers and contacts infected and genomic evidence confirming mink‐to‐human transmission. The cluster 5 variant (Y453F spike mutation) in Danish mink prompted culling of 17 million farmed mink [81]. Although the Y453F mutation increased the affinity for human ACE2, it did not substantially reduce neutralization by convalescent sera.

#### 3.6.3. H5N1 Mammalian Emergence

A joint FAO‐WOAH‐WHO risk assessment identified a single avian‐to‐bovine H5N1 spillover, followed by approximately 4 months of undetected cattle‐to‐cattle transmission [19]. Eisfeld et al. [82] demonstrated that bovine H5N1 can bind both avian (α2,3‐linked) and human (α2,6‐linked) sialic acid receptors, a critical prerequisite for efficient human‐to‐human transmission. Key mammalian adaptation markers include PB2 M631L [83] and additional PB2 and NP substitutions. Gu et al. [84] reported that a human isolate of bovine H5N1 is transmissible and lethal in ferret models. The intercontinental spread of clade 2.3.4.4b into North American dairy cattle and associated felids has been accompanied by rapid viral evolution [20, 85–87].

#### 3.6.4. Risk Hierarchy for New Reservoir Formation

SARS‐CoV‐2 has been detected in more than 60 animal species across dozens of countries [88, 89]. The risk hierarchy is as follows: farmed mink > white‐tailed deer > companion animals > other wildlife. The critical distinction is between species sustaining independent transmission cycles (deer and mink) versus dead‐end infections (most companion animals). Table 5 summarizes key species with confirmed natural SARS‐CoV‐2 infections, their inferred transmission directions, and supporting evidence. Collectively, the evidence indicates that reverse zoonosis has transitioned from a theoretical concern to a documented, quantifiable threat with immediate implications for pandemic control [88, 89].

**Table 5:** Key animal species with confirmed natural SARS‐CoV‐2 infections and inferred transmission directions.

Species (scientific name)	Taxonomic group	Geographic regions	Inferred transmission direction	Key evidence	References
White‐tailed deer (*Odocoileus virginianus*)	Cervid	USA, Canada	Human‐to‐animal; animal‐to‐animal; animal‐to‐human (spillback)	Deer‐adapted lineage B.1.641; deer‐to‐human transmission	[35, 36, 90]
American mink (*Neovison vison*)	Mustelid	Europe, North America	Human‐to‐animal; animal‐to‐animal; animal‐to‐human	Mink‐to‐human on farms; cluster 5 (Y453F)	[37, 81]
Syrian hamster (*Mesocricetus auratus*)	Rodent	Hong Kong	Animal‐to‐human	Delta (AY.127) hamster‐to‐human with onward transmission	[91]
Domestic cat (*Felis catus*)	Felid	Global	Human‐to‐animal	Natural infections in households and zoos; predominantly dead‐end	[88]
Domestic dog (*Canis lupus* familiaris)	Canid	Global	Human‐to‐animal	Natural infections; low seroprevalence	[88]
Lion (*Panthera leo*)	Felid	Zoos (global)	Human‐to‐animal	Confirmed infections in zoological collections	[88]
Tiger (*Panthera tigris*)	Felid	Zoos (USA)	Human‐to‐animal	Confirmed infections in zoological collections	[88]
Snow leopard (*Panthera uncia*)	Felid	Zoos (USA)	Human‐to‐animal	Confirmed infections in zoological collections	[88]
Western lowland gorilla (*Gorilla gorilla*)	Primate	Zoos (USA)	Human‐to‐animal	Confirmed infections in zoological collections	[88]
Ferret (*Mustela putorius* furo)	Mustelid	Domestic/laboratory	Human‐to‐animal	Natural and experimental infections; used as model	[88]
Multiple rodent species (pet trade)	Rodent	Global	Human‐to‐animal; potential animal‐to‐human	Pet‐shop‐associated outbreaks	[88, 89]

*Note:* Transmission directions are inferred from molecular epidemiology, contact tracing, and phylogenetic analyses. A comprehensive global registry of reported SARS‐CoV‐2 events in animals is provided by the SARS‐ANI dataset [89]; host‐range summaries are provided by Tan et al. [88].

Abbreviations: AA, animal‐to‐animal; AH, animal‐to‐human; HA, human‐to‐animal.

### 3.7. Intervention Effectiveness

#### 3.7.1. NPIs

Brauner et al. [33] estimated that limiting gatherings to ≤10 people reduced *R*_t_ by 42% (95% CI 29%–54%), closing schools and universities by 38% (95% CI 16%–54%), and stay‐at‐home orders provided only an additional 13% reduction beyond other already‐implemented measures (Figure 6). Flaxman et al. [38] estimated that NPI combinations reduced *R*_*t*_ by 82% across 11 European countries, though this model has been subject to methodological criticism [92].

**Figure 6:**
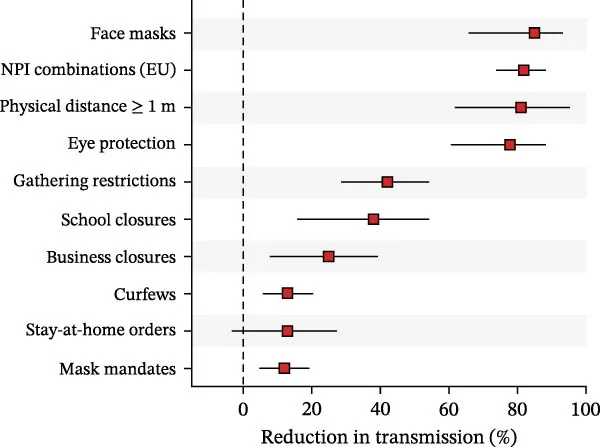
Forest plot of NPI effectiveness: *R*_*t*_ reduction by intervention type. Gathering restrictions (–42%) and school closures (–38%) show the largest effects; stay‐at‐home orders provide only marginal additional benefit (+13% beyond other NPIs). Data from Brauner et al. [33].

The lockdown evidence base is contested. Bendavid et al. [39] found no significant additional effect of more restrictive NPIs beyond less restrictive ones. Herby et al. [93] concluded that lockdowns reduced COVID‐19 mortality by only 2.0%–3.2% (up to 10.7% for specific NPIs). A Cochrane review rated the certainty of evidence for most physical interventions as low or very low [94]. Haug et al. [95] analyzed 6068 NPIs across 79 territories and found that less disruptive NPIs can be as effective as lockdowns. Our synthesis supports a graduated approach: targeted gathering restrictions and school closures as first‐line measures, with large‐scale lockdowns reserved for situations of epidemiological necessity where first‐line measures prove insufficient. Travel restrictions delayed the epidemic spread by days to weeks but did not prevent it [96].

An important caveat is that these effect estimates derive predominantly from high‐income settings during COVID‐19 [33, 38, 95]. Their rank ordering may not transfer directly to low‐ and middle‐income countries, where baseline contact patterns, household crowding, informal sector employment, and the feasibility of physical distancing differ fundamentally [18, 97]. In such settings, school closures may impose disproportionate social and nutritional harms while delivering smaller epidemiological benefits, and community‐level measures such as masking and ventilation may be relatively more attainable than household isolation. Comparative effectiveness across settings therefore remains a priority for future research.

#### 3.7.2. Vaccination

COVID‐19 vaccination showed high initial protection against infection, though its effectiveness declined over time, particularly against emerging variants [98]. Pivotal trials established high short‐term efficacy for the mRNA vaccines [99, 100]. Modeling studies demonstrated that maintaining moderate NPIs while scaling vaccination substantially reduced the projected disease burden [101]. Tsang et al. [102] demonstrated that pooled vaccine effectiveness against infection was 82% (95% CI 80%–83%) under high public health and social measure (PHSM) stringency versus 46% (95% CI 41%–50%) under low stringency, suggesting “leaky” vaccine characteristics that interact with population‐level transmission intensity. Mpox vaccination with JYNNEOS showed a two‐dose vaccine effectiveness of 66%–90% [103].

#### 3.7.3. Surveillance and Early Warning

Wastewater surveillance detected emerging SARS‐CoV‐2 variants approximately 1 week before clinical surveillance systems, including cryptic lineages not yet represented in clinical sampling [104]. AI‐enhanced surveillance systems showed promise for earlier outbreak detection through integration of multiple data streams, though data quality and interpretability remained challenges. Animal health surveillance systems remain a critical but underutilized component of early warning. The WOAH/FAO joint framework for the Progressive Control of Transboundary Animal Diseases (GF‐TADs) and the OFFLU influenza network provide established platforms for veterinary surveillance that can detect zoonotic threats at the animal–human interface. However, significant disparities persist in veterinary surveillance capacity: the WOAH Performance of Veterinary Services (PVS) pathway consistently identifies gaps in laboratory diagnostic capacity and epidemiological surveillance in low‐ and middle‐income countries.

#### 3.7.4. One Health Strategy and Cost‐Effectiveness

FAO estimated pandemic prevention costs at USD 10.3–11.5 billion per year versus USD 30.1 billion for management—a 3:1 cost advantage for prevention; investing USD 3 billion per year in One Health approaches could yield USD 37 billion in savings (ROI > 1100%) [105] (Table 6). Independent modeling corroborates that primary prevention of spillover is highly cost‐effective relative to the pandemic response [75, 76], and the One Health approach is increasingly formalized as the operational framework for such prevention [42, 43].

**Table 6:** Economic returns of One Health investment categories for zoonotic pandemic prevention.

Investment category	Description	Estimated ROI	Evidence basis
Upstream prevention	Wildlife surveillance, habitat protection, spillover prevention at source	≈1100% (10:1–11:1)	FAO cost‐benefit modeling; historical outbreak cost comparison
Integrated surveillance	Multisectoral human–animal–environment monitoring systems	≈500% (4:1–6:1)	GOHI‐Zoonoses framework; cross‐country performance data
Rapid response	Outbreak containment, contact tracing, ring vaccination	≈200% (2:1–3:1)	Ebola/COVID‐19 response cost‐effectiveness analyses
Vaccine R&D platforms	Pan‐coronavirus/pan‐filovirus prepandemic development	≈300% (2:1–5:1)	CEPI portfolio modeling; scenario analysis
Wastewater surveillance	Pathogen monitoring in wastewater systems	≈400% (3:1–5:1)	CDC NWSS cost savings; early detection value

*Note:* Values are modeled estimates based on FAO [105] and scenario analyses; actual returns vary by implementation context. The ROI figures rest substantially on a single FAO modeling exercise and should be interpreted with its underlying assumptions and uncertainty ranges in mind rather than as precise point estimates.

Abbreviations: CEPI, Coalition for Epidemic Preparedness Innovations; GOHI‐Zoonoses, Global One Health Index for Zoonoses; NWSS, national wastewater surveillance system; ROI, return on investment.

## 4. Discussion

Our synthesis yields three principal findings. First, zoonotic spillover is accelerating under anthropogenic pressures, with a 4.98% annual increase in high‐consequence events [46]. Land‐use change drives 50% of zoonotic diseases [69], yet pandemic preparedness investment remains predominantly reactive. The estimated >1100% ROI for One Health prevention [105] demands a fundamental reallocation of resources toward habitat conservation, agricultural biosecurity, and wildlife surveillance. The Plowright et al. [74] framework provides an operational roadmap: targeting the “spill” stage through land‐use planning and agricultural biosecurity may be more cost‐effective than downstream “spread” stage interventions.

Second, reverse zoonosis creates novel evolutionary risks that current surveillance systems are not designed to detect. The persistence of displaced SARS‐CoV‐2 variants in deer for >18 months after human extinction [80] and the undetected months‐long transmission of H5N1 in dairy cattle [19, 20] demonstrate that animal‐adapted viruses can evolve independently and re‐emerge in human populations. Surveillance must be expanded to include key reservoir species, particularly farmed mink, free‐ranging deer, and intensively managed livestock. The accelerated evolution of SARS‐CoV‐2 in deer [79] implies that animal reservoirs may generate antigenically novel variants capable of evading population immunity acquired through vaccination or prior human infection. One Health genomic surveillance integrating human, animal, and environmental sampling is essential [40, 41].

Third, the evidence supports targeted, proportional intervention over blunt population‐wide measures. The effectiveness hierarchy—gathering restrictions and school closures at 38%–42% *R*_*t*_ reduction, with minimal marginal benefit from lockdowns at ~13% [33]—argues for precision public health approaches targeting specific transmission pathways. The NPI–vaccine synergy [101] and leaky vaccine characteristics under varying NPI stringency [102] support sequential deployment where NPIs buy time for vaccine development and scale‐up, followed by proportionate relaxation as population immunity increases.

### 4.1. Limitations

Several limitations warrant emphasis. First, statistical heterogeneity was substantial (*I* ^2^ frequently >90%), reflecting genuine biological and methodological diversity across pathogens, settings, and study designs; although random‐effects models accommodate this, pooled point estimates should be interpreted as weighted summaries rather than precise parameters. Second, publication and reporting bias are plausible given the disproportionate research volume devoted to COVID‐19, and trim‐and‐fill adjustments can only partially correct for the missing studies. Third, geographic representation was uneven, with an over‐representation of high‐income countries and a corresponding under‐representation of the tropical regions where many zoonotic reservoirs are concentrated. Fourth, several headline quantities—notably H5N1 CFR [47] and the One Health economic returns [105]—depend on detection‐biased case ascertainment or on modeled projections with wide uncertainty ranges, respectively. Fifth, cross‐pathogen comparisons are confounded by heterogeneous surveillance infrastructure, as acknowledged in Section 1. These caveats do not undermine the central conclusions but define the confidence bounds within which they should be applied.

## 5. Conclusions

The 2016–2026 decade witnessed an acceleration of emerging viral zoonotic events that is both statistically robust and operationally significant. Three strategic implications emerge: (a) upstream prevention is systematically underfunded relative to downstream response—the >1100% ROI for One Health prevention [105] demands resource reallocation toward habitat conservation and wildlife surveillance; (b) reverse zoonosis creates novel evolutionary risks requiring expanded genomic surveillance in key reservoir species, with particular attention to farmed mink, cervids, and intensively managed livestock [80, 88]; and (c) targeted, proportional intervention outperforms blunt population‐wide measures in both effectiveness and societal cost [33, 95].

Specifically, the veterinary sector must be fully integrated into pandemic preparedness architectures through the investment in veterinary diagnostic networks, wildlife disease surveillance programs, and livestock biosecurity measures. The WOAH PVS Pathway should be leveraged as a framework for building national One Health capacities, and animal health surveillance data must be made interoperable with human health systems through shared platforms such as the Global Early Warning System for Emerging Animal Diseases (GLEWS+). The accelerating pace of zoonotic spillover, compounded by reverse zoonosis and synergistic anthropogenic drivers, demands a structured One Health response integrating ecological surveillance, targeted intervention, and economic optimization [40, 42]. The cost of inaction—measured not only in lives lost but in the expanding universe of viral evolutionary possibilities created by animal reservoirs—far exceeds the investment required for prevention [76].

## Author Contributions

**Pan Tao**: conceptualization, methodology, formal analysis, investigation, writing – original draft. **Ying-an Zang**: investigation, data curation, formal analysis. **Jing Wang**: investigation, validation. **Feng Cong**: conceptualization, supervision, writing – review and editing, project administration. **Ming Liao**: conceptualization, resources, writing – review and editing, supervision, funding acquisition.

## Funding

This research was supported by the Guangzhou Municipal Science and Technology Project (Grant 2025A04J0379), the Guangdong Provincial Special Project in Key Fields for General Higher Education Institutions (Grant KA26YY15030), and the Zhongkai Scholars Program (Grant KA26YY00106‐2026).

## Disclosure

All authors have read and approved the final manuscript. The funders had no role in study design, data collection and analysis, decision to publish, or preparation of the manuscript.

## Conflicts of Interest

The authors declare no conflicts of interest.

## Data Availability

All data extracted for this systematic review and meta‐analysis are available from the corresponding author upon reasonable request.
